# Cognitive Priming During Warmup Enhances Sport and Exercise Performance: A Goldilocks Effect

**DOI:** 10.3390/brainsci15030235

**Published:** 2025-02-23

**Authors:** Jesús Díaz-García, Ana Rubio-Morales, David Manzano-Rodríguez, Tomás García-Calvo, Christopher Ring

**Affiliations:** 1 Department of Psychology, University “G. d’Annunzio”, 66013 Chieti-Pescara, Italy; jesus.diaz@unich.it; 2 BIND-Behavioral Imaging and Neural Dynamics Center, University “G. d’Annunzio”, 66013 Chieti-Pescara, Italy; 3 Faculty of Sport Sciences, University of Extremadura, 10003 Caceres, Spain; 4 School of Sport, Exercise & Rehabilitation Sciences, University of Birmingham, Birmingham B15 2TT, UK

**Keywords:** fatigue, performance, sleep, warmup

## Abstract

**Background:** Mental fatigue can impair sport, exercise and cognitive performance. Warmup activities can improve performance when the individual is rested. However, their effectiveness when the individual is fatigued has yet to be established. The research objectives were to evaluate the effects of physical and combined physical plus cognitive warmup activities on subsequent sport, exercise, and cognitive performance when rested and fatigued by sleep restriction in athletes (Study 1) and older adults (Study 2). **Methods:** In Study 1, 31 padel players completed a padel performance test and Stroop task after physical and combined warmups when rested and fatigued by sleep deprivation. In Study 2, 32 older adults completed sit–stand, arm curl, walking, Stroop, and psychomotor vigilance tests after no warmup, physical warmup, and combined warmup when rested and fatigued by sleep deprivation. In both studies, combined warmups intermixed short-, medium-, or long-duration cognitive tasks between physical warmup activities. Mental fatigue was measured using visual analog scale ratings. **Results:** In both studies, sleep deprivation increased mental fatigue and impaired performance. In Study 1, relative to a physical warmup, padel and Stroop performance were improved by combined warmups (with short-to-medium cognitive tasks) when rested and fatigued. In Study 2, relative to no warmup, sit–stand, arm curl, walking, Stroop, and reaction time performance were improved by physical and combined warmups (with short-to-medium cognitive tasks) when rested and fatigued. **Conclusions:** The negative effects of sleep deprivation on sport, exercise, and cognitive performance were best mitigated by combined warmups with short-to-medium cognitive tasks. Combined warmups are effective countermeasures against the deleterious effects of mental fatigue on performance.

## 1. Introduction

Mental fatigue, defined as an acute state of tiredness and diminished brain functioning, can impair sport and exercise performance [1,2,3]. A state of increased mental fatigue can be assessed using increased subjective ratings of mental fatigue, impaired cognitive task performance, and changes in physiological activity, such as reduced heart rate variability [4,5]. Interviews with athletes and their support personnel confirm that mental fatigue, a common problem experienced by elite athletes [6], can be elicited by a variety of personal and situational demands, including sleep loss associated with participation in tournaments and early/late training sessions [7,8,9]. Preventive physiological and psychological countermeasures have been developed to overcome the problem of mental fatigue [10]. To our knowledge, no study has evaluated preparatory warming up activity as a mental fatigue countermeasure and performance enhancer.

Despite an absence of empirical evidence, there is anecdotal evidence that some athletes use a combination of cognitive and physical activities before they compete in their sport. For instance, racing drivers may warm up for 15 min before the start of a race with a mixture of cognitive (e.g., decision-making and reaction time drills) and physical (e.g., muscular and cardiovascular drills) activities [11]. Researchers have argued that lightboard drills, which typically impose relatively low cognitive demands, can be used by athletes as part of a neuromuscular warmup protocol to activate the central nervous system and thereby prepare athletes for upcoming sport-related stimulus and response information processing demands [12]. However, to date, there is a lack of empirical studies providing an evidence base to design effective warmup protocols. Accordingly, the current two study research project sought to address this gap in our understanding of ways to counteract the detrimental effects of mental fatigue induced by sleep deprivation on performance.

Sleep deprivation is defined as incidental or intentional lack of sleep (i.e., less than 7 h sleep within 24 h) due to artificial manipulations or natural situations [13]. Sleep deprivation can impair exercise and sport performance [14]. For example, studies show that sleep deprivation can impair weightlifting exercises [15], tennis serving accuracy [16], and padel reaction times [17]. Reviews of the literature confirm that sleep deprivation can impair basic cognitive performance, including processing speed and executive functions [18,19], with these cognitive impairments evident in a variety of professions, such as service personnel [20] and athletes [14]. To deal with the negative impact of sleep deprivation on performance, various countermeasures have been evaluated [21], including taking rest breaks, consuming stimulant drugs, and using neural stimulation devices. The utility of warming up activities has not, to our knowledge, been systematically investigated in this context.

Individuals are encouraged to physically warm up before they start to play sport or exercise. This is because passive and active warmup activities can help improve subsequent sport and exercise performance [22,23,24]. For instance, a study of young soccer players found that sprinting and jumping performance were improved immediately following 15 min of physical warmup activities (e.g., jogging, stretching, joint mobility, sprinting, jumping) compared to when a 15-min rest separated the warmup activities and physical performance tests or when there was no warmup [25]. This study also found that cognitive performance, assessed by reaction times on a 10-min psychomotor vigilance test (PVT) completed after the exercise tests, was better when the physical warmup was not followed by rest or when there was no warmup. These findings were replicated and extended in a related study involving football referees, which found that sprinting and PVT performance were better when the warmup was not followed by a rest [26]. A later study of student athletes reported that 15 min of physical warmup activities improved subsequent basketball dribbling performance compared to no warmup [27]. Interestingly, in this latter study, participants completed the 10-min PVT before the 15-min physical warmup. Accordingly, the improved basketball dribbling performance may be attributed, at least in part, to the earlier cognitive task, perhaps by the combined warming up tasks preparing them to perform (e.g., via perceptual–motor priming).

A priming explanation is compatible with research showing that performing brief cognitive tasks can improve subsequent videogame performance in young adults [28], cognitive task performance in children [29], and cognitive task performance in older adults [30]. There are various forms of cognitive priming, including perceptual priming [31] and response priming [32], that can improve subsequent task performance. Similarly, mental skill warmup activities (e.g., arousal regulation, goal setting, imagery, self-talk) can improve subsequent readiness to perform [33,34] and sport and exercise performance [35]. However, the possibility that adding a brief cognitive task, such as the PVT, to standard physical warmup activities can improve subsequent performance is contradicted by studies showing that performing long-duration cognitive tasks, such as the Stroop color–word task, can impair subsequent sport and exercise performance [1,2,3]. In sum, there is good evidence that physical warmups improve subsequent performance; however, the effects of adding cognitive activities to warmup protocols have yet to be established. Accordingly, the current research studies have sought to explore this priming phenomenon.

Having identified a gap in current understanding of countermeasures to mitigate any effects of sleep deprivation and mental fatigue on performance, we designed two studies to evaluate the effects of physical warmups, with and without intermixed cognitive activities, on subsequent sport, exercise, and cognitive performance. Given that the effects of mental fatigue and sleep deprivation on performance may be moderated by fitness and age [36], we evaluated the effects of warmups on performance in both athletic young adults (Study 1) and sedentary older adults (Study 2).

### Study 1

Study 1 evaluated the effects of adding different duration cognitive tasks to standard physical warmup activities on subsequent padel and cognitive performance following standard sleep (i.e., when rested) and sleep deprivation (i.e., when fatigued) in young adult athletes. Our first research purpose was to compare the effects of physical warmup and combined physical plus cognitive warmup on sport and cognitive performance. We hypothesized that combined warmup would improve or impair padel shot performance and cognitive response inhibition compared to the standard physical warmup. Based on past studies (see above), we expected that performance would be improved by adding short cognitive tasks to physical warmups but impaired by adding long cognitive tasks to physical warmups. Our second research purpose was to compare the effects of standard and deprived sleep on sport performance, cognitive performance and mental fatigue. We hypothesized that sleep deprivation would increase mental fatigue and impair both padel shot accuracy and cognitive response inhibition compared to standard sleep.

## 2. Method

### 2.1. Participants

Thirty-one (16 males, 15 females) U23 high-level padel players who trained five times per week and played in official tournaments of the Spanish National Federation were recruited from local clubs and gave informed consent. Players were approached in person by an experimenter who provided information and answered questions about the study. This study was conducted according to the guidelines of the Declaration of Helsinki and approved by the Ethics Committee of the University of Extremadura (93, 2020). Power calculations [37] indicated that with a sample of 31, the study was powered at 80% to detect significant (*p* < 0.05) within-participant effects corresponding to small-to-medium effect sizes (2 levels: η_p_^2^ = 0.06, f = 0.26; 4 levels: η_p_^2^ = 0.04, f = 0.21) by repeated measures analysis of variance (ANOVA).

### 2.2. Mental Fatigue

Visual Analogue Scale—Mental Fatigue (VAS-MF): Participants were asked to indicate how mentally fatigued they felt by marking a 10-cm line, with anchors of “not at all” and “maximum level of mental fatigue possible” [38].

### 2.3. Cognitive Performance

Brief Stroop Task: A 45 s brief Stroop task performed on a smartphone (UMH-MEMTRAIN, Elche, Spain) assessed response inhibition, a core executive function operation [39]. Participants were shown a series of color-incongruent words and were instructed to name the color of the word. The number of words that participants spoke out loud was recorded. Researchers told participants when their answer was incorrect; they responded until correct. Performance was measured as the total number of correct words spoken.

### 2.4. Sport Performance

The Padel Stroke Performance Test [40] assessed padel shot accuracy. Participants performed a set of four shots of each of four padel strokes (drive, drive volley, bandeja, drive after-glass attack), with a 20 s rest separating the sets. The ball was propelled by a machine to standardize ball delivery. Shot accuracy was scored by two courtside assessors using a point-based scoring system; the closer the returned ball bounced to the corner of the court, the more points were awarded. For instance, the most and least accurate shots were awarded 3 and 0 points, respectively. Mean performance accuracy scores were computed for each padel stroke.

### 2.5. Sleep Status Conditions

Participants were tested in two sleep status conditions: following a night of standard sleep (rested) and following a night of sleep deprivation (fatigued). Participants slept in their own bed at home. Based on a week’s self-reported sleep habits, the deprivation was created by asking participants to go to bed at a specific delayed time and to wake up at the usual time so that the total amount of sleep corresponded to 60% of standard sleep duration.

### 2.6. Warmup Conditions

We created four warmup conditions: a standard physical warmup condition and three combined warmup conditions that comprised a standard physical warmup intermixed with relatively short-, medium-, and long-duration cognitive tasks. The standard physical warmup comprised a series of exercises: 10-min jogging, 7-min 30-s dynamic stretching, 7-min 30-s resistance band movements, and 7-min 30 s low-intensity padel-specific movements on court (turns, lateral running, squats, jumps). Each of the four exercises was followed by a 2-min 30 s recovery involving passive stretching. Each recovery was immediately (i.e., without rest) followed by nothing or one of three Stroop task durations: 2 min 30 s, 4 min 23 s, or 7 min 30 s. Thus, the four warmup conditions comprised physical activity lasting a total of 32.5 min intermixed with cognitive activity lasting a total of 0 min, 10 min, 17.5 min, and 30 min (i.e., cognitive task duration). The Stroop task [39] was performed on a laptop computer (UMH-MEMTRAIN, Elche, Spain). This executive function task assesses response inhibition. A color word (blue, green, red, yellow) was displayed in a different color on a black background (1900 ms interstimulus interval). Participants were told to press a button to indicate the meaning of the words as quickly and accurately as possible.

### 2.7. Procedure

**Figure 1:**
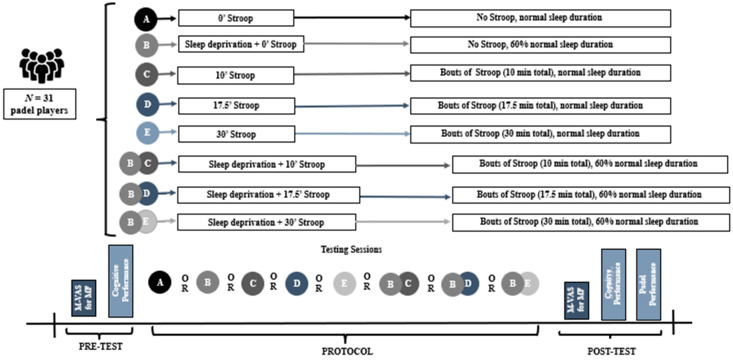
Study 1 protocol.

The study employed a within-participant experimental design, with cognitive task duration (0, 10, 17.5, 30 min) and sleep status (rested, fatigued) as within-participant factors (Figure 1). Participants completed one familiarization session followed by eight testing sessions. Each session was completed on a different day at approximately the same time of day. Participants were encouraged to refrain from caffeine and alcohol for 12 h before each session. Testing session order was randomized. In the familiarization session, participants reported their standard sleep habits, provided a VAS-MF rating, practiced both Stroop tasks, and performed a padel test. In each of the eight testing sessions, they provided a VAS-MF rating, completed a brief Stroop task, performed a warmup, provided a VAS-MF rating, completed a brief Stroop task, and performed a padel test. These activities were completed sequentially without a rest period between them. Four of the testing sessions were completed the day after standard sleep and four were completed the day after sleep deprivation.

### 2.8. Data Analysis

A series of ANOVAs with warmup cognitive task duration (0, 10, 17.5, 30 min) and sleep status (rested, fatigued), as within-participant factors were performed on the measures of padel shot accuracy. A series of mixed-factorial ANOVAs with warmup cognitive task duration (0, 10, 17.5, 30 min), sleep status (rested, fatigued) and state (before warmup, after warmup) as within-participant factors were performed on the mental fatigue ratings and number of correct words in the brief Stroop task. *T*-tests directly compared levels and/or factors following significant effects (*p* < 0.05). To reduce the risks of violating ANOVA’s sphericity and compound symmetry assumptions, the multivariate solution was provided. The effect size [41], partial eta squared (η_p_^2^), was provided, with values of 0.02, 0.13, and 0.26 reflecting small, medium, and large effects, respectively. Statistical analysis was performed using SPSS 29.

## 3. Results

### 3.1. Sport Performance

**Figure 2:**
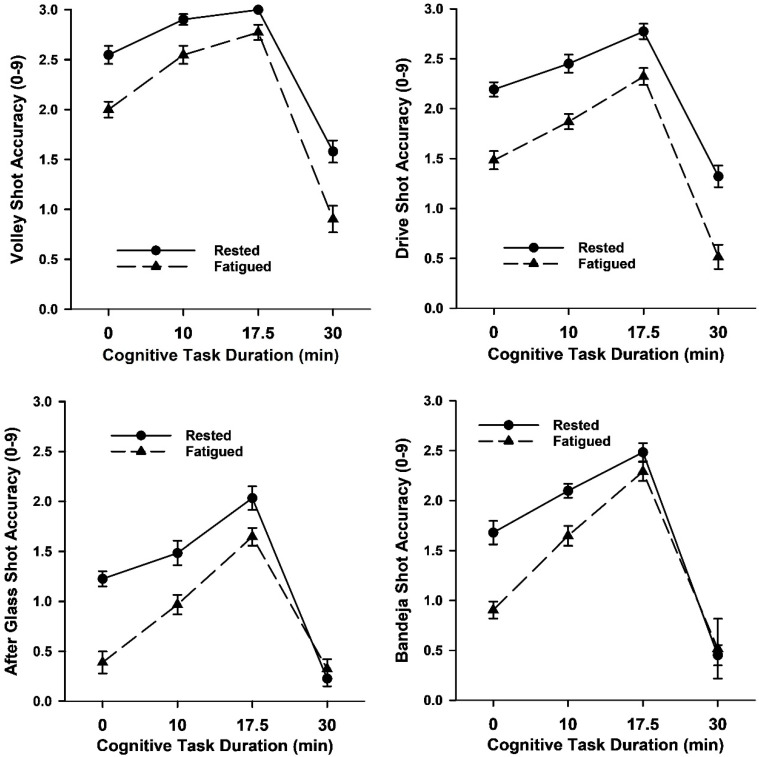
Mean (*SE*) shot accuracy during the padel stroke performance test as a function of warmup cognitive task duration and sleep status in Study 1.

A series of 4 warmup cognitive task duration (0, 10, 17.5, 30 min) by 2 sleep status (rested, fatigued) ANOVAs on padel shot accuracy yielded the main effects on task duration and sleep status (Figure 2 and Table 1). Compared to the standard physical warmup (i.e., 0-min cognitive duration), padel performance when rested and fatigued was improved by the 10-min and 17.5-min cognitive durations intermixed with the physical warmup but impaired by the 30-min cognitive duration. Players returned the ball less accurately when fatigued by sleep deprivation than when rested. Interaction effects were found for two of the strokes. After-glass shot accuracy was better when rested than fatigued for the 0-min, 10-min and 17.5-min task durations but not for the 30-min task duration. Bandeja shot accuracy was better when rested than fatigued for the 0-min and 10-min durations but not the 17.5-min and 30-min durations.

### 3.2. Mental Fatigue

**Figure 3:**
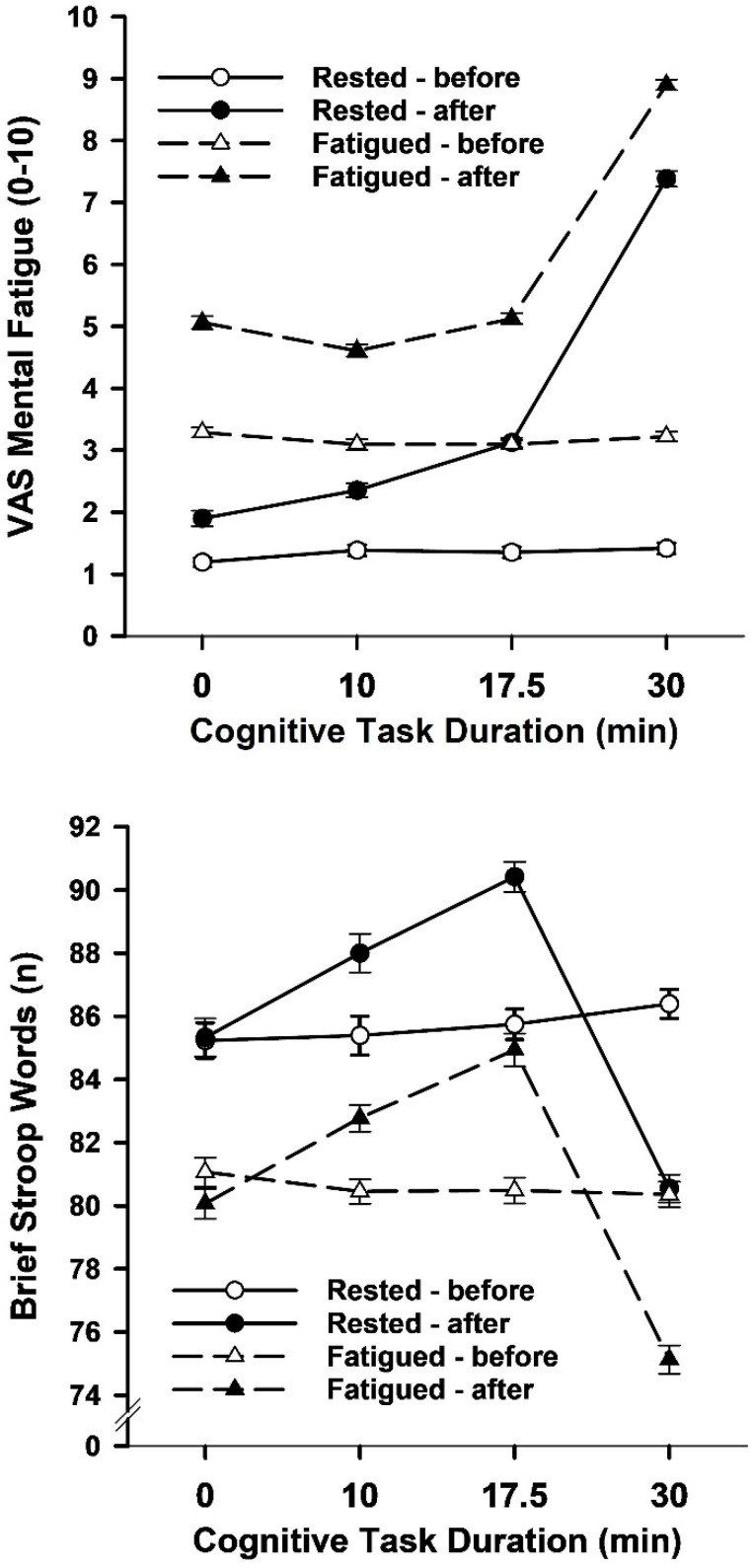
Mean (*SE*) mental fatigue ratings and brief Stroop task correct responses as a function of warmup cognitive task duration, sleep status and test timing in Study 1.

The 4 cognitive duration (0, 10, 17.5, 30 min) by 2 sleep status (rested, fatigued) by 2 state (before warmup, after warmup) ANOVA on mental fatigue ratings found main and interaction effects (Figure 3 and Table 2). The main effects for cognitive duration, sleep status, and state showed that mental fatigue ratings were higher for the longest cognitive duration compared to the shorter durations, higher after sleep deprivation (fatigued) than standard sleep (rested), and higher after than before the warmup, respectively. The key duration by sleep by state interaction effect confirmed that when rested following standard sleep and fatigued by sleep deprivation the mental fatigue ratings were stable before the warmup whereas after the warmup the fatigue ratings were higher for the 30-min duration compared to the 0-min, 10-min and 17.5-min durations.

### 3.3. Cognitive Performance

The 4 cognitive duration by 2 sleep status by 2 state ANOVA of the number of correct words vocalized during the brief Stroop task produced main and interaction effects (Figure 3 and Table 2). The cognitive duration, sleep status, and state main effects indicated that cognitive performance was better after short-to-medium duration warmups and worse after long-duration warmups than no warmup, worse when fatigued than rested, and somewhat better after than before the combined warmup. Importantly, the key three-way interaction effect confirmed that performance was stable before the warmup when rested and fatigued, whereas performance varied after the warmup depending on the duration of the warmup, with more words vocalized after the 17.5 min warmup than the 10-min warmup, more words after the 10-min warmup than no warmup, and more words after no warmup than the 30-min warmup.

## 4. Discussion

Study 1 evaluated the effects of adding cognitive tasks to a standard physical warmup on subsequent padel shot accuracy and cognitive response inhibition when both rested and fatigued by sleep deprivation. Compared to the standard warmup with no cognitive tasks, subsequent padel shot accuracy and response inhibition was facilitated by intermixing 2.5-min and 4.375-min cognitive tasks with physical warmup exercises but impaired by intermixing 7.5-min cognitive tasks. Sleep deprivation amounting to 60% standard sleep duration consistently impaired the sport and cognitive performance of high-level padel players. Moreover, the abovementioned pattern was observed when players were feeling relatively rested (after a standard night’s sleep) and fatigued (after a shortened night’s sleep). These findings provide novel evidence that combined warmups with small-to-medium doses of cognitive activities can improve subsequent sport and cognitive performance and mitigate the deleterious effect of fatigue on sport and cognitive performance caused by sleep deprivation. However, a large dose of cognitive activity impaired subsequent sport and cognitive performance to levels below those observed without warming up when fatigued by sleep deprivation. This phenomenon can be considered a *Goldilocks effect* (i.e., just the right amount; see [42]) for cognitive warmup activities before playing sport and performing executive function operations. These findings suggest that there is an optimal dose of cognitive warmup activities to enhance subsequent sport and cognitive performance. It is likely that this “*just right*” dose will depend on a variety of factors, including the nature of the activity, the experience of the individual, and the structure of the physical warmup routine. In sum, we found evidence showing that intermixing brief (<5 min) cognitive activities with sets of standard physical warmup routines are effective countermeasures against the deleterious effects of mental fatigue on performance.

### Study 2

Building on Study 1’s findings, Study 2 compared the effects of no warmup, physical warmup, and combined physical and cognitive warmup activities on subsequent exercise and cognitive performance following standard sleep and sleep deprivation in sedentary older adults. Our third research purpose was to compare the effects of no, physical, and combined warmups on resistance exercise, endurance exercise, cognitive executive function, and cognitive non-executive function performance. We hypothesized that combined warmup would improve exercise (lower and upper body resistance, aerobic endurance) and cognitive (response inhibition, attention) performance compared to physical warmup, and, in turn, physical warmup would improve performance compared to no warmup. Our fourth research purpose was to compare the effects of standard sleep and sleep deprivation on exercise and cognitive performance and mental fatigue. We hypothesized that sleep deprivation would increase mental fatigue and impair exercise and cognitive performance compared to standard sleep.

## 5. Method

### 5.1. Participants

Thirty-two (16 males, 16 females) sedentary (no regular exercise activity) older adults (range 68–77, *M* = 71.47, *SD* = 2.14 years) were recruited from the local community and gave informed consent. The adults were approached in person by an experimenter who provided information and answered questions about the study. The exclusion criteria were aged less than 65 years, physical or cognitive training in the last three years, and mental or physical medical conditions that impaired the cognitive and exercise tasks. This study was conducted according to the guidelines of the Declaration of Helsinki and approved by the Ethics Committee of the University of Extremadura (93, 2020). Power calculations indicated that with a sample of 32, the study was powered at 80% to detect significant (*p* < 0.05) within-participant effects corresponding to a small-to-medium effect sizes (2 levels: η_p_^2^ = 0.06, f = 0.26; 3 levels: η_p_^2^ = 0.05, f = 0.23) by repeated measures ANOVA.

### 5.2. Mental Fatigue

Mental fatigue was assessed using a VAS-MF rating (see Study 1).

### 5.3. Exercise and Cognitive Performance

Sit–Stand Test: The 30-s sit-stand test [43] measured lower-body strength. Participants sat on a chair and crossed their arms in front of their chest and placed their hands over their shoulders. From this starting position, they repeatedly stood up (with full knee extension) and sat down again as many times as possible in 30 s. The number of sit–stand repetitions was recorded.

Arm Curl Test: The 30-s arm curl test [44] measured upper body strength. Participants sat on a chair and held a 5 kg dumbbell in their dominant hand. From this starting position, they repeatedly curled their elbow until it was fully flexed (touching their shoulder) and slowly lowered the dumbbell as many times as possible in 30 s. A successful repetition was counted for each complete movement. The number of arm curl repetitions was recorded.

Brief Stroop Task: A brief 45-s Stroop task was used to assess response inhibition. Participants were shown a series of color-incongruent words performed on a smartphone (UMH-MEMTRAIN, Elche, Spain), and the number of correct words they vocalized was recorded. Performance was measured as the total number of correct words spoken.

Brief Psychomotor Vigilance Task (PTV-B): A 3-min PVT-B [45] was used to assess attention (i.e., readiness to respond). A visual stimulus, with a 1–4-s variable interstimulus interval, was shown in the center of a smartphone screen. Participants were required to press the touch-sensitive screen as fast as possible. Performance was measured as reaction time (ms).

Walking Test: The 6-min walk test [46] measured endurance exercise performance. Participants walked up and down a flat 30 m corridor. They walked as far as possible in 6 min. The distance (m) walked was recorded. Upon completion, participants gave a rating of perceived exertion (RPE) regarding the previous exercise on a 0–10 scale with anchors of “minimal” and “maximal” [47].

### 5.4. Sleep Status Conditions

Two sleep status conditions (rested, fatigued) were created per Study 1, except that the sleep deprivation (fatigued) corresponded to 50% of standard sleep duration (rested).

### 5.5. Warmup Conditions

We created three warmup conditions: no warmup, physical warmup, combined physical and cognitive warmup. The no warmup was a control and involved no activities. The physical warmup comprised a series of exercises: 5-min brisk walking, 5-min dynamic stretching, 5-min resistance band movements, and 5-min brisk walking, with each of the four exercises preceded and followed by a 2.5-min period involving passive stretching. Thus, the physical warmup comprised physical activity lasting a total of 20 min intermixed with stretching activity lasting a total of 12.5 min. The combined physical and cognitive warmup involved the same physical exercises, with each of the four exercises immediately (i.e., without rest) preceded and followed by a 2.5-min cognitive task (Stroop task; see Study 1). Thus, the combined warmup comprised physical activity lasting 20 min intermixed with cognitive activity lasting 12.5 min.

### 5.6. Procedure

**Figure 4:**
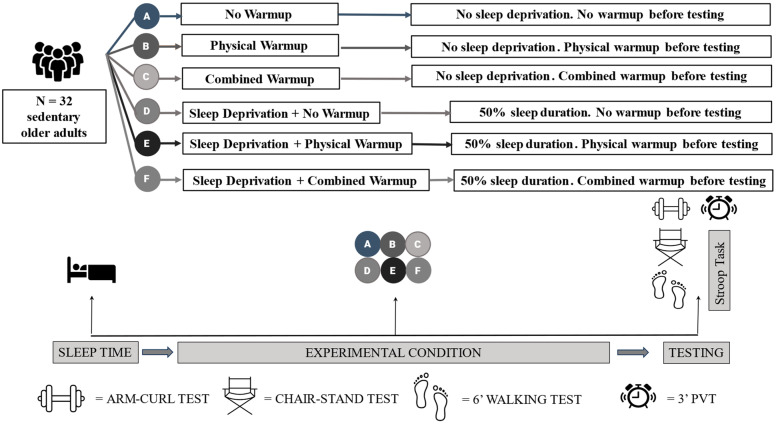
Study 2 protocol.

The study employed a within-participant experimental design, with warmup (no, physical, combined) and sleep status (rested, fatigued) as within-participant factors (Figure 4). Participants completed one familiarization session followed by six randomized testing sessions. Each session was completed on a different day at approximately the same time of day. Participants were encouraged to refrain from caffeine and alcohol for 12 h before each session. In the familiarization session, they reported their standard sleep habits, completed ratings, and practiced the exercise tests and cognitive tasks. In each of the six testing sessions (three rested, three fatigued), they completed the warmups, ratings and tests/tasks in the following order: warmup, VAS-MF, sit-stand test, arm curl test, Stroop task, PVT-B task, walking test, and RPE. These activities were completed sequentially without a rest period between them.

### 5.7. Data Analysis

Adopting a similar analytic strategy to Study 1, ANOVAs with warmup (no, physical, combined) and sleep status (rested, fatigued) as within-participant factors were performed on the exercise, exertion, fatigue and cognitive measures.

## 6. Results

### 6.1. Exercise Performance

**Figure 5:**
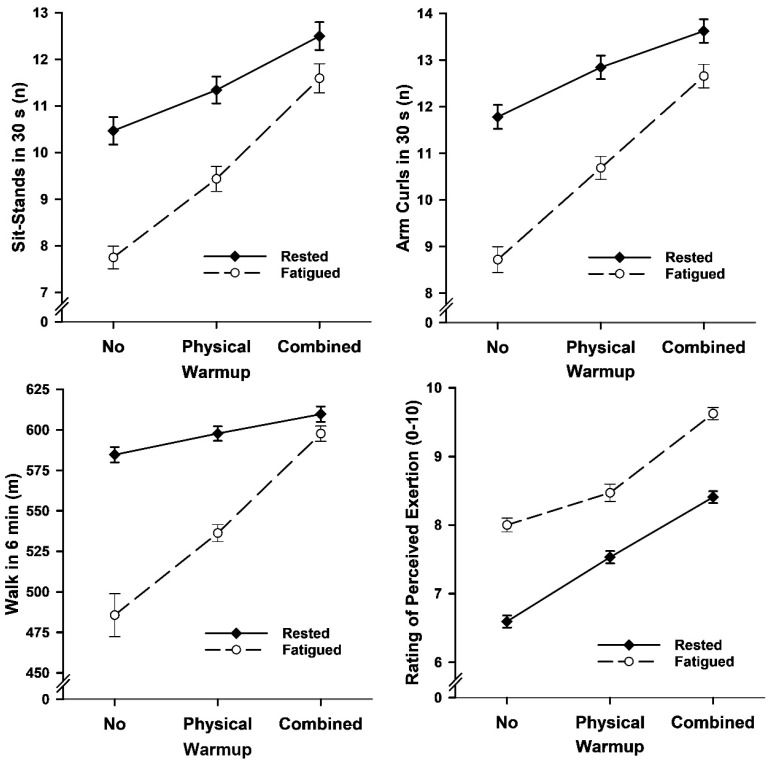
Mean (*SE*) exercise test performance and perceived exertion as a function of warmup and sleep status in Study 2.

The 3 warmup (no, physical, combined) by 2 sleep status (rested, fatigued) ANOVAs on exercise performance yielded warmup main effects (combined warmup better than physical warmup, and physical warmup better than no warmup), sleep status main effects (rested better than fatigued), and warmup by sleep status interaction effects for all three tests (Figure 5 and Table 3). The interaction effects showed that the rested-fatigued difference in performance was least with combined warmup and most with no warmup; this performance difference was less with combined warmup than physical warmup and less with physical warmup than no warmup. ANOVA on RPE confirmed effects for warmup (combined warmup higher than physical warmup, and physical warmup higher than no warmup), sleep status (fatigued higher than rested), and warmup by sleep status (rested–fatigued difference most for no warmup and least for physical warmup).

### 6.2. Mental Fatigue

**Figure 6:**
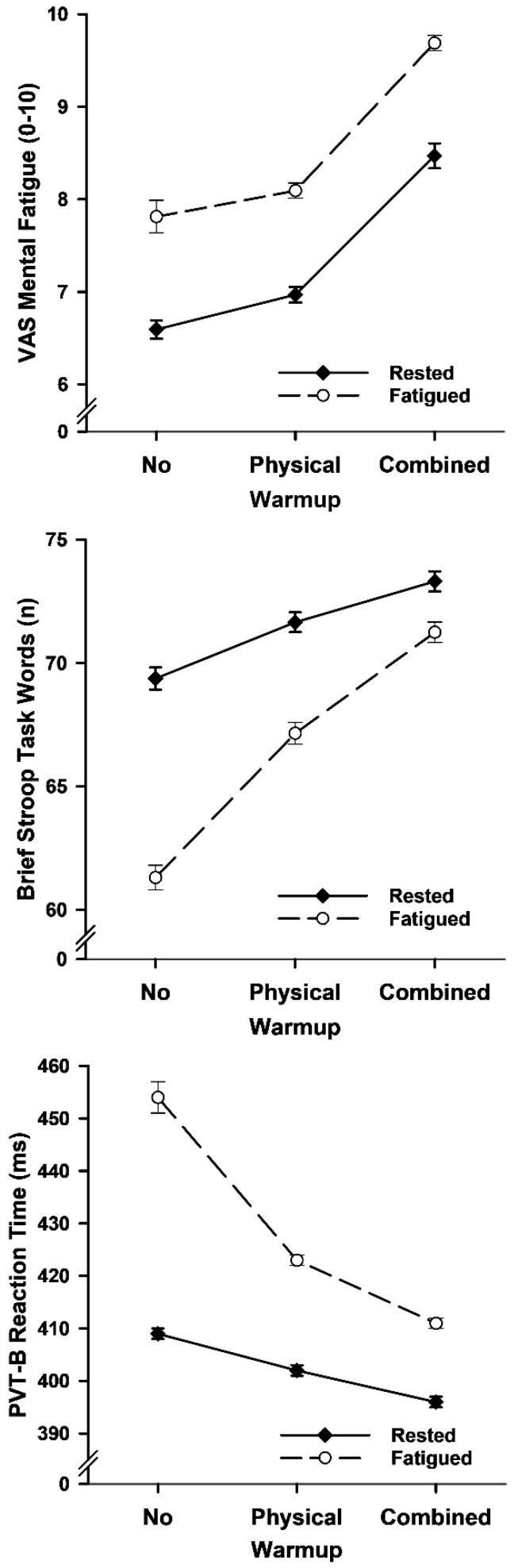
Mean (*SE*) mental fatigue ratings, brief Stroop task correct responses, and PVT-B reaction times as a function of warmup and sleep status in Study 2.

The 3 warmup by 2 sleep status ANOVAs on mental fatigue ratings found main effects for warmup and sleep status (Figure 6 and Table 3). VAS-MF ratings were lower for no warmup than physical warmup, lower for physical warmup than combined warmup, and lower following standard sleep (i.e., rested) than sleep deprivation (i.e., fatigued).

### 6.3. Cognitive Performance

The 3 warmup by 2 sleep status ANOVAs on cognitive task performance yielded main and interaction effects for reaction times and correct words (Figure 6 and Table 3). Task performance was better for combined warmup than physical warmup, better for physical warmup than no warmup, and better when rested than fatigued. Moreover, the rested–fatigued performance difference was greater for no warmup than physical warmup, and greater for physical warmup than combined warmup.

## 7. Discussion

Study 2 evaluated the effects of physical, combined and no warmup activities on subsequent resistance exercise, endurance exercise, cognitive response inhibition, and cognitive vigilance (sustained attention) when both rested and fatigued by sleep deprivation in older adults. Regarding our third research purpose, we found that exercise and cognitive performance was improved by warmup activities compared to no warmup activities, and, moreover, this performance enhancement effect was greater for combined physical plus cognitive warmups than physical only warmups. Regarding our fourth research purpose, we found that sleep deprivation amounting to 50% standard sleep duration consistently impaired exercise and cognitive performance. Importantly, the pattern of performance enhancement associated with warmup activities revealed that the deterioration in exercise and cognitive performance following sleep deprivation was mitigated by the warmups, with the greatest mitigation following combined physical warmup activities. In sum, we found evidence for the effectiveness of warmups as mental fatigue countermeasures.

## 8. General Discussion

We explored the effects of warmup type and duration—comprising brief physical tasks performed either alone or combined with brief cognitive tasks—on subsequent sport, exercise and cognitive performance when feeling mentally fatigued after a truncated night’s sleep or rested after a standard night’s sleep. We found that combined warmups that incorporated short-to-medium duration cognitive tasks (i.e., from 10–17.5 min) optimized subsequent sport performance (Study 1), exercise performance (Study 2), and cognitive performance (Studies 1 and 2) when participants were mentally fatigued by sleep deprivation or rested following standard sleep. We also found that combined warmups partly mitigated the negative effects of mental fatigue, caused by sleep deprivation, on performance. Accordingly, the current findings reveal that warming up activities act as countermeasures against the deleterious effects of mental fatigue associated with sleep deprivation on performance. Our key findings are discussed in detail below.

Our first research purpose was to compare the effects of physical warmup and combined physical plus cognitive warmup on sport and cognitive performance in athletes. Study 1 revealed that performing Stroop tasks after standard physical warmup activities, such as jogging and stretching, improved subsequent padel shot accuracy when the individual Stroop tasks lasted 150 s (total duration 10 min) and 263 s (total duration 17.5 min) but impaired shot accuracy when they lasted 450 s (total duration 30 min) compared to the physical warmup alone. These findings are compatible with past research showing that a combined warmup comprising a 10-min cognitive task before 15-min of physical activities improved basketball dribbling more than the 15-min physical warmup alone [27]. However, these findings contrast with those of a study that reported no effects of combined warmups up on a running-based psychomotor test [48]. The finding that the segmented long duration cognitive task during the warmup was then followed by impaired sport performance is compatible with evidence that continuous long duration demanding cognitive tasks alone can impair subsequent sport performance [1,2,3]. For example, a continuous 30-min Stroop task impaired subsequent padel shot speed and accuracy [49]. These findings imply that players should include short but not long bouts of cognitive tasks during warmups to benefit subsequent racquet sport performance. Moreover, they should monitor the overall cognitive dose (i.e., duration by intensity) to regulate the evoked state of relative mental vigor versus fatigue, with the former being beneficial for performance and the latter being detrimental for performance.

Study 1 also found that the combined warmups with 10-min and 17.5-min of cognitive tasks improved subsequent cognitive performance more than the physical warmup alone whereas the combined warmups with 30-min of cognitive tasks impaired subsequent cognitive performance relative to the physical warmup alone. We are not aware of any similar previous research studies to directly compare with these findings. It is worth noting that past studies report that successfully performing brief cognitive tasks can improve subsequent cognitive task performance [30,50]. Similar benefits have been reported for videogame performance [28]. It is possible that warmup activities, by performing cognitive tasks as part of a combined warmup protocol, transfer positively to subsequent basic cognitive tasks as well as sport-specific tasks with core cognitive elements. These speculations warrant empirical examination.

Our second research purpose was to compare the effects of standard and deprived sleep on sport performance, cognitive performance and mental fatigue in athletes. Study 1 found that sleep deprivation increased mental fatigue and impaired sport and cognitive performance. Moreover, adding short bouts of a cognitive Stroop task, totaling 10 min or 17.5 min, to normal physical warmups acted as a countermeasure to combat the negative effects of sleep deprivation on padel performance. These results suggest that the negative effects of sleep loss on task performance may be mediated by parallel impairments in cognitive operations. Previous studies report that sleep deprivation impairs arousal and attention, both of which are key to good cognitive performance [51]. Indeed, these researchers demonstrated that caffeine, a popular mental fatigue countermeasure, counteracts sleep deprivation. Similarly, Study 1 found that short-to-medium (but not long) cognitive warmups counteracted sleep deprivation and its deleterious effects on sport and cognitive performance. Since previous studies have reported that competing in padel tournaments incrementally decreases the amount of sleep and increases the state of mental fatigue [7,17] athlete support personnel, such as coaches, should monitor their players’ sleep and fatigue and explore whether their deleterious effects on performance can be mitigated by combined warmup activities.

Our third research purpose was to compare the effects of no warmup, physical warmup and combined warmup on resistance exercise, endurance exercise, cognitive executive function, and cognitive non-executive function performance in older adults. Study 2 provides novel evidence in favor of the physical and cognitive warmup activities acting as countermeasures to tackle aging-related declines in performance. Specifically, we documented the effectiveness of both physical and combined warmups on older adults’ subsequent exercise and cognitive performance, when feeling fresh and fatigued by lack of sleep. To the best of our knowledge, this is the first study to document this phenomenon in older adults. Specifically, we found that having older adults complete a brief combined (i.e., physical and cognitive) warmup improved their performance of submaximal exercise tests assessing endurance (6-min walk test), resistance-based leg strength endurance (sit-stand test), and upper body strength endurance (arm curl test). The combined warmup also improved their performance of cognitive tasks assessing readiness to respond (PVT-B task) and response inhibition (Stroop color–word task). These improvements were evident under testing conditions when the older adults were both rested and fatigued (sleep deprivation). Although physical warmup also improved performance compared to control (no warmup), our findings revealed that the benefits were less than those seen with the combined warmup. These findings for exercise performance are in line with those for sport performance reported in Study 1.

Our fourth research purpose was to compare the effects of standard sleep and sleep deprivation on exercise and cognitive performance in older adults. With regard to the effects of sleep deprivation on older adults’ physical and cognitive performance, Study 2’s findings confirm previous evidence showing that sleep deprivation impairs performance. As noted above, sleep deprivation exacerbates aging-related impairments in cognitive and physical performance [52,53].

### 8.1. Study Limitations and Future Directions

The present studies yielded important new findings on the benefits of warmups for performance. However, their interpretation should consider potential methodological limitations. First, the studies assessed the effects of warmups on performance of a small number of sport, exercise and cognitive tasks. Future research should examine a larger number and broader range of tasks to determine the generalizability of the phenomena reported here. Second, the warmups included only one cognitive activity—the Stroop color–word task. Studies should explore the effects of other response inhibition tasks, tasks that involve other executive functions such as memory updating, switching, sustained attention and decision-making, and tasks that involve non-executive functions such as perception and information processing speed. They should also explore the benefits of using batteries of tasks during warmup rather than just the one task in order to warmup a broader range of cognitive operations and increase novelty and interest for participants to enhance engagement. Third, we did not corroborate sleep quantity (or quality). Future investigations could use wearables to provide objective measures of sleep. Fourth, the sample sizes were relatively small. Accordingly, the current findings should be replicated using larger, more diverse samples. Fifth, we adopted within-participant designs. Although we randomized the order of testing to mitigate the negative effects of repeated testing on performance (e.g., carryover and practice effects), future studies should replicate the current findings using between-participant designs.

### 8.2. Practical Implications

In terms of practical guidelines, our study findings may help coaches and trainers to tackle the effects of sleep deprivation and associated increased state of mental fatigue on performance. We offer some practical recommendations for individuals and support personnel. The first recommendation is to monitor sleep and mental fatigue when training and/or competing in events and competitions. For those purposes, the Pittsburgh Sleep Quality Index, for sleep, and the Visual Analogue Scale, for mental fatigue, are recommended. The second recommendation is to include short bouts of cognitive activities intermixed with physical activities, such as stretching and jogging, before performing any test or task. By incorporating short cognitive tasks into warmup routines, coaches could enhance athlete readiness and subsequent sport performance. The present studies show that combined warmups improve performance when feeling fresh and rested after sleeping sufficiently, but also largely mitigate the deleterious effects of sleep deprivation. Individuals can do this by including short bouts of cognitive tasks using apps running on smart devices, as the present studies did, but also, they might be able to use a more specific cognitive task, such as a drill where coaches’ instructions are linked to sport-specific actions or movements. Similar drills could be developed for older adults training at home or in the gym.

## 9. Conclusions

Our studies revealed the additive benefits of supplementing standard physical activities with brief cognitive activities for subsequent sport, exercise and cognitive performance. This novel evidence suggests that combined and intermixed physical and cognitive warmups can help improve human performance across a broad range of ages (young to older adults), experiences (fit athletes to sedentary non-athletes), and domains (sport, exercise, cognition). These exciting findings provide the preliminary empirical evidence to encourage individuals and trainers to adapt their warmup protocols to better prepare players for competition and seniors for workouts.

## Figures and Tables

**Table 1:** Summary of the 4 task duration (0, 10, 17.5, 30 min) by 2 sleep status (rested, fatigued) ANOVAs on sport performance in Study 1.

Measure	Task Duration		Sleep Status		Task Duration × Sleep Status	
	***F*(12,19)**	**η_p_^2^**	***F*(4,27)**	**η_p_^2^**	***F*(12,19)**	**η_p_^2^**
Volley accuracy	134.39 ***	0.82	49.88 ***	0.74	2.97	0.09
Drive accuracy	154.16 ***	0.84	84.26 ***	0.62	1.82	0.06
After-glass accuracy	89.84 ***	0.75	39.89 ***	0.57	7.48 *	0.20
Bandeja accuracy	66.81 ***	0.69	7.45 **	0.20	4.35 *	0.13

Notes. *** *p* < 0.001; ** *p* < 0.01; * *p* < 0.05.

**Table 2:** Summary of the 4 task duration (0, 10, 17.5, 30 min) by 2 sleep status (rested, fatigued) by 2 state (before warmup, after warmup) ANOVAs on mental fatigue and cognitive performance in Study 1.

Measure	Task Duration		Sleep Status		Testing Time		Task Duration × Sleep Status		Task Duration × Testing Time		Sleep Status × Testing Time		Task Duration × Sleep Status × Testing Time	
	*F*(6,180)	η_p_^2^	*F*(2,29)	η_p_^2^	*F*(2,29)	η_p_^2^	*F*(6,180)	η_p_^2^	*F*(6,180)	η_p_^2^	*F*(2,29)	η_p_^2^	*F*(6,180)	η_p_^2^
VAS mental fatigue	475.10 ***	0.94	1296.30 ***	0.98	3744.76 ***	0.99	24.82 ***	0.45	604.55 ***	0.95	15.75 ***	0.34	110.27 ***	0.27
Stroop words	160.68 ***	0.84	236.60 ***	0.89	13.78 ***	0.32	1.84	0.06	972.66 ***	0.97	3.91	0.12	7.55 ***	0.20

Notes. *** *p* < 0.001; VAS = visual analog scale.

**Table 3:** Summary of the 3 warmup (no, physical, combined) by 2 sleep status (rested, fatigued) ANOVAs on exercise performance, perceived exertion, mental fatigue, and cognitive performance in Study 2.

Measure	Condition		Sleep Status		Condition × Sleep Status	
	***F*(2,30)**	**η_p_^2^**	***F*(1,31)**	**η_p_^2^**	***F*(2,30)**	**η_p_^2^**
Sit–stand repetitions	152.63 ***	0.91	506.10 ***	0.94	83.01 ***	0.85
Arm curl repetitions	152.50 ***	0.91	1660.28 ***	0.98	60.62 ***	0.80
Walking distance	425.45 ***	0.97	2218.83 ***	0.99	193.58 ***	0.93
RPE	209.72 ***	0.93	170.42 ***	0.85	4.47 *	0.23
VAS mental fatigue	182.03 ***	0.92	315.73 ***	0.91	0.34	0.02
Stroop words	297.91 ***	0.95	487.98 ***	0.94	51.66 ***	0.78
PTV-B reaction time	516.31 ***	0.97	924.53 ***	0.97	142.41 ***	0.91

Notes. *** *p* < 0.001; * *p* < 0.05. RPE = rating of perceived exertion. VAS = visual analog scale.

## Data Availability

The raw data supporting the conclusions of this article will be made available by the authors on request. The data are not publicly available due to privacy restrictions.
